# Composite Assessment Using Intestinal Ultrasound and Calprotectin Is Accurate in Predicting Histological Activity in Ulcerative Colitis: A Cohort Study

**DOI:** 10.1093/ibd/izad043

**Published:** 2023-03-16

**Authors:** Thomas M Goodsall, Alice S Day, Jane M Andrews, Andrew Ruszkiewicz, Christopher Ma, Robert V Bryant

**Affiliations:** IBD Service, Department of Gastroenterology, John Hunter Hospital, Newcastle, Australia; Faculty of Health Sciences, School of Medicine, University of Adelaide, Adelaide, Australia; Faculty of Health Sciences, School of Medicine, University of Adelaide, Adelaide, Australia; IBD Service, Department of Gastroenterology, The Queen Elizabeth Hospital, Adelaide, Australia; Faculty of Health Sciences, School of Medicine, University of Adelaide, Adelaide, Australia; IBD Service, Department of Gastroenterology, The Queen Elizabeth Hospital, Adelaide, Australia; Surgical Pathology Division, SA Pathology, Adelaide, Australia; Division of Gastroenterology and Hepatology, Department of Medicine, University of Calgary, Calgary, Alberta, Canada; Department of Community Health Sciences, University of Calgary, Calgary, Alberta, Canada; Faculty of Health Sciences, School of Medicine, University of Adelaide, Adelaide, Australia; IBD Service, Department of Gastroenterology, The Queen Elizabeth Hospital, Adelaide, Australia

**Keywords:** ulcerative colitis, inflammatory bowel disease, intestinal ultrasound, histology, fecal calprotectin

## Abstract

**Background:**

Beyond endoscopic remission, histological remission in ulcerative colitis (UC) is predictive of clinical outcomes. Intestinal ultrasound (IUS) may offer a noninvasive surrogate marker for histological activity; however, there are limited data correlating validated ultrasound and histological indices.

**Aim:**

Our aim was to determine the correlation of IUS activity in UC with a validated histological activity index.

**Methods:**

Twenty-nine prospective, paired, same-day IUS/endoscopy/histology/fecal calprotectin (FC) cases were included. Intestinal ultrasound activity was determined using the Milan Ultrasound Criteria, histological activity using the Nancy Histological Index, endoscopic activity using Mayo endoscopic subscore and Ulcerative Colitis Endoscopic Index of Severity, and clinical activity using the Simple Clinical Colitis Activity Score.

**Results:**

Histological activity demonstrated a significant linear association with overall IUS activity (coefficient 0.14; 95% CI, 0.03-0.25; *P* = .011). Intestinal ultrasound activity was also significantly associated with endoscopic activity (0.32; 95% CI, 0.14-0.49; *P* < 0.001), total Mayo score (0.31; 95% CI, 0.02-0.60; *P* = .036) but not FC (0.10; 95% CI, −0.01 to 0.21; *P* = .064) or clinical disease activity (0.04; 95% CI, −0.21 to 0.28; *P* = .768). A composite of IUS and FC showed the greatest association (1.31; 95% CI, 0.43-2.18; *P* = .003) and accurately predicted histological activity in 88% of cases (*P* = .007), with sensitivity of 88%, specificity 80%, positive predictive value 95%, and negative predictive value 57%.

**Conclusions:**

Intestinal ultrasound is an accurate noninvasive marker of histological disease activity in UC, the accuracy of which is further enhanced when used in composite with FC. This can reduce the need for colonoscopy in routine care by supporting accurate point-of-care decision-making in patients with UC.

**Key Messages**

**What is already known?** Intestinal ultrasound is accurate in identifying intestinal inflammation in ulcerative colitis and Crohn’s disease compared with endoscopic activity. It is well-tolerated, noninvasive, and available at the point of care. The accuracy of intestinal ultrasound for assessing histological activity is not well-described.

**What is new here?** This pilot cohort study demonstrated a strong association between a composite of IUS activity/fecal calprotectin and histological activity in ulcerative colitis.

**How can this study help patient care?** Intestinal ultrasound and fecal calprotectin may be used as noninvasive markers for activity in ulcerative colitis and enhance point-of-care decision-making while reducing the need for endoscopic assessment.

## Introduction

Ulcerative colitis (UC) can be associated with substantial symptom burden, reduced quality of life, and risk of colectomy for severe disease or neoplasia.^1^ The expanding availability of effective medical therapies coupled with tight monitoring in a treat-to-target paradigm has raised therapeutic expectations. STRIDE guidelines advise that endoscopic remission should now be the therapeutic goal in UC, as it has been associated with reduced risk of flare, hospitalization, and surgery.^2,3^ However, even when endoscopic mucosal healing is achieved, histological inflammation persists in one-third of patients.^4^ Amelioration of histological inflammation better predicts risk of UC relapse, corticosteroid use, and hospitalization than endoscopic remission alone.^4,5^ Therefore, there is increasing support for histology to also be used as a potential treatment target in UC as a more complete measure of mucosal healing.^3,6^

Despite the utility of histological remission and normalization as predictors of disease course in UC, acquisition of colonic mucosal biopsies for histological assessment necessitates endoscopy, which is costly, burdensome for patients, and associated with procedural risk. Furthermore, the lack of standardization of histological reporting has limited clinical adoption. Of thirty described indices, very few have been validated for accuracy, reliability, and responsiveness.^7^ The Nancy Index and Robarts Histological Index (RHI) have most recently been developed and robustly validated in UC.^8,9^

The capacity of surrogate indices to replace the necessity for invasive tests in UC is appealing given the increasing drive for tight monitoring and repeated measurements as a part of a treat-to-target paradigm. Fecal calprotectin correlates well with histological remission, and the area under the receiver operating curve ranges from 0.63 to 0.95.^10^ Disadvantages of FC include false positive rates for inflammation, high intra-individual and interindividual variability, performance dependent on assay and cutoff used, and an inability to determine disease extent, severity, or presence of complications.^11–13^

Intestinal ultrasound (IUS) is a useful, noninvasive and well-tolerated method of accurately assessing colonic and small bowel inflammation without the need for fasting, bowel preparation, or sedation.^14–17^ Active inflammation is characterized by thickening of the bowel wall (BWT), intramural vascularity on color Doppler imaging, and changes in bowel wall stratification.^15^ Intestinal ultrasound has been shown to accurately determine disease activity and correlates well with endoscopic mucosal activity, with sensitivity of 71% to 100% and specificity of 64% to 100%.^14,15,17–19^ The unique capacity of IUS to visualize both mucosal and transmural inflammatory activity raises the possibility that it may be a useful surrogate marker for histological activity. Provisional data demonstrate an association between IUS and histological activity; however, studies to date have been small and limited by the absence of validated indices for either histological and/or IUS, which lends itself to the likelihood of unconscious and unaddressed bias and limits generalizability and reproducibility—of special importance to clinical trial applications.^20–23^

The aim of this study was therefore to determine the correlation of IUS activity with a validated histological activity index, fecal calprotectin, endoscopic disease activity, and clinical activity in UC using prospective same-day multi-assessments.

## Methods

### Participants

Participants were a subgroup of the EAT-UC study, a prospective cohort study of dietary intervention in UC.^24^ Participants with a formal diagnosis of UC underwent same-day IUS, calprotectin, and colonoscopy with biopsy at study entry. Intestinal ultrasound, calprotectin, and colonoscopy with biopsy were repeated at 8 weeks after a dietary intervention. Ethical approval was granted by the Central Adelaide Local Health Network Ethics Committee (HREC/16/RAH/24, R20160202). All participants with paired IUS and colonoscopy/histology findings were included.

### Intestinal Ultrasound

Intestinal ultrasound was performed (Canon Aplio a550 18LX5 probe, Doppler velocity 5 cm/s) by a single expert sonographer who was blinded to the results of colonoscopy (R.V.B.) as previously described.^25^ BWT was measured as the perpendicular distance from the echogenic luminal interface to the outer muscularis boarder on a cross-sectional image. Bowel wall vascularity was assessed using color Doppler imaging (CDI) and considered present if persistent luminal signal was identified during breath holding. Bowel wall stratification (BWS) was recorded as normal, abrogated, or lost. Ulcerative colitis disease activity was scored using the Milan Ultrasound Criteria (MUC), which applies a linear algorithm of BWT and CDI to give a continuous activity score where active disease includes a score of >6.3 (Appendix 1).^26,27^ The most affected region of the sigmoid colon was recorded for correlation with biopsy location and histology. A subset of IUS studies (*n* = 24) stored as DICOM files were verified by a blinded central reader (T.M.G.) to confirm data accuracy.

### Colonoscopy and Biopsy

Colonoscopy was performed by an IBD specialized gastroenterologist who was blinded to the results of IUS. Colonoscopy was performed under sedation following a standard split dose bowel preparation with glycoprep. On completion of colonoscopy, the Mayo endoscopic subscore and Ulcerative Colitis Endoscopic Index of Severity (UCEIS) score were prospectively recorded. Active endoscopic disease was defined as a Mayo endoscopic score of ≥1.^2^ Endoscopic disease extent was also recorded according to Montreal criteria.^28^ Two biopsies were taken from the most affected bowel segment, or in cases without endoscopic activity from the sigmoid colon and rectum, and fixed in formalin 10% for histological analysis.

### Histological Assessment

Histological assessment was performed by an expert pathologist who was blinded to the clinical information and results of IUS and colonoscopy. Histology was scored using the Nancy Histological Index (NHI)^8,29^ The NHI was selected due to simplicity of reporting characteristics, validation, and demonstration of excellent intra- and interobserver agreement (Appendix 2).^9,30^ Histological remission was classified as an NHI score of ≤1 according to published cutoffs.^8,29^

### Clinical and Biomarker Disease Activity

Clinical disease activity was determined by the Simple Clinical Colitis Activity Score (SCCAI) and Partial Mayo Score, categorized using published thresholds.^31^ The clinical and endoscopic Mayo subscores were also used to calculate the total Mayo score. Fecal calprotectin (FC) was recorded for each patient using a sample collected within 48 hours of endoscopic examination (before bowel preparation) and a cutoff value of 100 ug/g used to distinguish active disease.^12^

### Study Outcomes

The primary outcome was correlation of IUS UC activity with histological activity. Secondary outcomes were correlation of individual IUS parameters (BWT, CDI, BWS) with histological disease activity, endoscopic disease activity, and clinical disease activity.

### Statistical Analysis

Statistical analysis was performed using Stata (v14, StataCorp, Tx, USA). Correlation between all outcomes was determined using a linear effects model to adjust for repeated observations over time, after confirming assumptions of a linear model. Fecal calprotectin was transformed using a log conversion for linear analysis. Categorical comparisons were performed using the Fisher exact test (2-sided). The McNemar test was used to determine whether the composite end point of IUS activity and calprotectin was superior to Milan Ultrasound Criteria alone.

## Results

Paired IUS and colonoscopy/histology data were available for 29 cases in 19 patients. The demographic and clinical information is listed in Table 1. The full spectrum of disease activity was represented in the Mayo score distribution. Nancy Histological Index grading was distributed across the full spectrum of activity.

**Table 1.** Demographic information for 19 participants of the EAT-UC trial with paired IUS, endoscopy and histology outcomes.

Age, years		46 (21-72)
Gender, female/male		11/8
Disease extent	E1 (proctitis)	0
	E2 (Left sided)	11
	E3 (Extensive)	8
Mayo score	Remission (0-2)	4 (14)
	Mild^3–5^	8 (28)
	Moderate^6–9^	14 (48)
	Severe^10–12^	3 (10)
SCCAI	Remission (0-2)	3 (10)
	Mild^3–5^	16 (55)
	Moderate^6–11^	10 (35)
	Severe^12–19^	0
UCEIS	Remission (0-1)	5 (17)
	Mild^2–4^	13 (45)
	Moderate^5,6^	11 (38)
	Severe^7,8^	0
NHI	0	3 (10)
	1	2 (7)
	2	11 (38)
	3	8 (28)
	4	5 (17)
MUC		6.2 (2.1-13.3)
Bowel wall Thickness, mm		3.0 (1.5-8.1)
Fecal Calprotectin, ug/g		190 (4-1808)
Albumin (g/L)		39 (33-44)

Data are median (range), or *n* (%) values.

Abbreviations: SCCAI, Simple Clinical Colitis Activity Index; UCEIS, Ulcerative Colitis Endoscopic Index of Severity; NHI, Nancy Histological Index; MUC, Milan Ultrasound Criteria

### Correlation of Histology With Intestinal Ultrasound Activity

**Figure 1.**
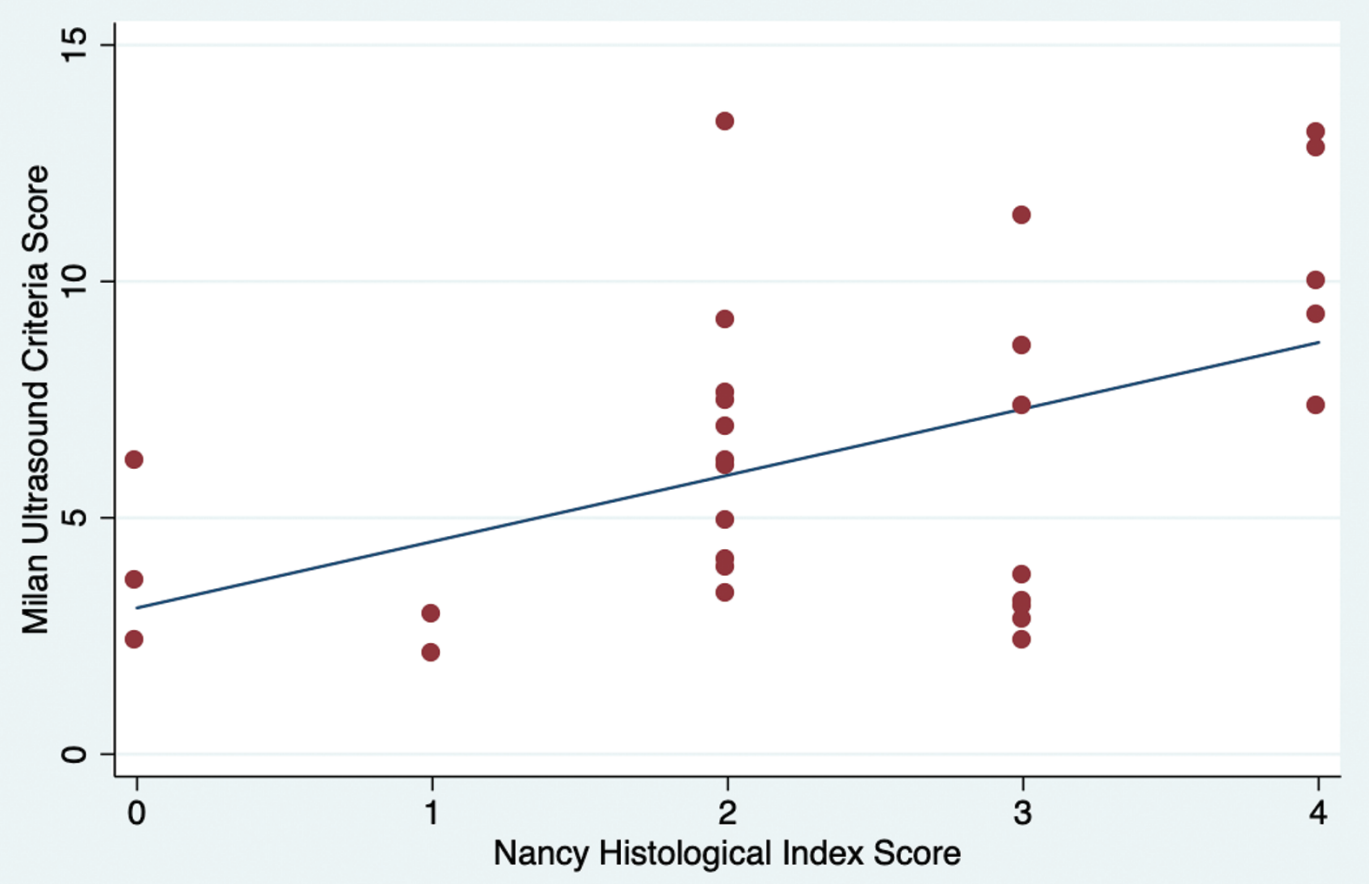
Scatter plot of Nancy Histological Index Score and Milan Ultrasound Criteria with line of best fit.

There was a significant linear association between the NHI histological score and overall IUS activity using the MUC (coefficient 0.14; 95% CI, 0.03-0.25; *P* = .011; Figure 1 and Table 2). Bowel wall thickness alone was significantly associated with NHI histological activity (coefficient 0.29; 95% CI, 0.07-0.50; *P* = .009), although CDI was not (coefficient 0.65; 95% CI, −0.07 to 1.39; *P* = .077). When a composite IUS and FC activity rating was assigned as active if either FC ≥100 ug/g or IUS MUC >6.3, the strength of linear association with NHI increased (coefficient 1.307; 95% CI, 0.43-2.18; *P* = .003).

**Table 2.** Linear association of ulcerative colitis activity indices.

Comparison	Coefficient	95% Confidence Interval	*P*
NHI and MUC	0.14	0.03-0.25	.011
NHI and BWT	0.29	0.07-0.50	.009
NHI and CDI	0.66	−0.07 to 1.39	.077
NHI and Calprotectin	0.27	0.01-0.53	.044
NHI and composite of MUC and calprotectin^a^	1.3	0.43-2.18	.003
MUC and Mayo Score	0.31	0.02-0.60	.036
MUC and SCCAI	0.04	−0.21 to 0.28	.768
MUC and UCEIS	0.32	0.14-0.49	<.001
MUC and Calprotectin	0.10	−0.01 to 0.21	.064

^a^MUC and fecal calprotectin composite determined as active if MUC >6.3 and/or fecal calprotectin ≥100 ug/g. Abbreviations: SCCAI, Simple Clinical Colitis Activity Index; UCEIS, Ulcerative Colitis Endoscopic Index of Severity; NHI, Nancy Histological Index; MUC, Milan Ultrasound Criteria

When histological and IUS activity were treated as binary (ie, remission or active, remission NHI ≤1 and MUC ≤6.3 respectively), there was a significant association (*P* = .048 by Fisher exact test). In all 5 cases of histological remission, the IUS MUC score was <6.3. In the 24 cases where histological activity was present, the IUS MUC score was >6.3 in 13 (55%). Intestinal ultrasound sensitivity for histological activity was 55%, specificity 100%, positive predictive value 100%, and negative predictive value 31%. When a composite IUS and FC activity rating was assigned as active if FC >100 ug/g and/or IUS MUC >6.3, the association remained significant (*P* = .007) and was significantly greater than IUS MUC alone using the McNemar test (*P* = .004). The composite score’s sensitivity for histological activity (88%) was greater than the association of FC with histological activity alone (79%; Table 3 and Appendix 3).

**Table 3.** Categorical association and operating characteristics of measures of disease activity compared with histological activity (NHI > 1).

Comparison	*P*	Sensitivity (%)	Specificity (%)	Positive Predictive Value (%)	Negative Predictive Value (%)
MUC >6.3	.048	55	100	100	31
Calprotectin >50 ug/g	.127	92	40	88	50
Calprotectin >100 ug/g	.022	79	80	95	44
Composite of MUC and calprotectin^a^	.007	88	80	95	57

*P* value determined by Fisher exact test. ^a^MUC and fecal calprotectin composite determined as active if MUC >6.3 and/or fecal calprotectin ≥100 ug/g.

Abbreviations: NHI, Nancy Histological Index; MUC, Milan Ultrasound Criteria

### Correlation of IUS With Endoscopic and Clinical Activity

Intestinal ultrasound activity demonstrated a significant linear association with the total Mayo score (coefficient 0.307; 95% CI, 0.020-0.595; *P* = .036) and UCEIS (coefficient 0.32; 95% CI, 0.14-0.49; *P* < 0.001) but not the SCCAI (coefficient 0.04; 95% CI, −0.21 to 0.28; *P* = .768). There was a significant linear association between log-converted FC and histological activity using the Nancy index (coefficient 0.27; 95% CI, 0.01-0.53; *P* = .044) but not IUS activity using the MUC (coefficient 0.10; 95% CI, −0.01 to 0.21; *P* = .064; Table 2).

## Discussion

This study provides new understanding of the correlation between IUS and histological activity using validated indices to measure clinical, endoscopic, histologic, and biomarker disease activity. The significant linear association between IUS and the NHI and high (100%) positive predictive value of IUS support its use in a treat-to-target management paradigm in UC. The correlation and sensitivity of IUS can be enhanced using a composite IUS/FC determinant of activity. Point-of-care IUS is appropriate for tight monitoring of UC in that it is well-tolerated, preferred by patients, and can facilitate immediate decision-making.^15,32^

The association between IUS and histological activity has been previously described in studies that have been limited by sample size, methodology, or lack of validated indices and limited generalizability. The first study by Bozkurt et al demonstrated that patients with IBD or infective colitis had a significantly greater proportion of biopsies demonstrating histological activity in cases where IUS demonstrated increased bowel wall thickness and loss of bowel wall stratification.^33^ Haber et al also described a significant correlation between histological activity and both bowel wall thickness and loss of bowel wall stratification in IBD.^21^ Watanabe et al found that bowel wall thickness assessed with endoscopic ultrasound correlated with histological grade in acute severe UC.^23^ Drews et al demonstrated a significant association between IUS measured bowel wall vascularity and histological activity in Crohn’s disease.^20^ More recently, Sagami et al reported good correlation of transperineal ultrasound activity in the rectum and histological indices of activity (Geboes, RHI, and Nancy indices), the only study to use validated histological indices.^22^

Although several small studies have compared IUS to histological activity in UC, few/none have used a validated index. The validated NHI was used in this study; however, the NHI lacks the ability to distinguish histological remission and normalization. These terms have been variably defined in the literature to date. Many studies define histological remission as the absence of active inflammation such as neutrophilic infiltration, crypt abscesses, or erosions.^7,9^ Histological normalization is characterized by normal crypt architecture in addition to the features of remission.^7,9^ Histologic normalization may occur with treatment in 10% of cases of UC and has been associated with a decreased risk of clinical relapse compared with histological remission.^34,35^ Whether IUS is capable of distinguishing these states is unclear, although it is possible that sonographic prominence and thickening of the submucosal layer may represent a harbinger of histological inflammation in UC.

Standardization of IUS disease activity assessment in UC is limited. Bowel wall thickness is the most accurate and reproducible measure and several composite scores have been proposed by various combinations of bowel wall thickness with color Doppler vascularity, changes in bowel wall stratification, mesenteric inflammatory fat, or presence of reactive lymph nodes.^19^ The MUC is one of several IUS activity indices proposed for UC and was selected for ease of use, external validation, and favorable operating characteristics.^19,26,27,36^ Although IUS activity indices offer theoretical benefits in assessing the magnitude of disease activity for clinical trials and responsiveness, a simple outcome is most beneficial in most clinical practice scenarios, and BWT alone demonstrated significant linear association with histological activity. Further studies using a validated IUS activity index are critical to better define disease activity, response to treatment, and disease remission if IUS is to be used in clinical trials.

This study offers a prospective comparison of simultaneous, same-day IUS and endoscopic/histological data with FC collected within 48 hours. Although FC and IUS individually perform well when determining disease activity in UC, it is the combination of FC and IUS in this study that overcomes the individual test limitations and offers a powerful, noninvasive, composite proxy for histological activity. One benefit of the composite end point is in capturing those cases of distal rectal inflammation for which transabdominal IUS has a lower sensitivity. The blinded reading of histology and IUS activity also reduced risk of bias. Strict targets of endoscopic remission (Mayo endoscopic score of 0) and biochemical remission were selected in accordance with STRIDE II consensus statements.^3^ There are some limitations to this study. Histological activity does not always result in sonographically detectable activity, and even when using a composite end point, IUS cannot accurately exclude histological activity in every patient. However, the use of surrogate measures of inflammation is never perfect but must be balanced against cost, patient acceptability, and application in clinical care to achieve close monitoring. The sample size was small and derived from a preexisting single-center study group selected by a trial inclusion criteria, so not prospectively powered to address the current question with the full range of disease activity. However, the quality of generated data was maximized through the blinded reading of histology and IUS. Some observations were repeated studies in the same patient at 0 and 8 weeks, which necessitated the use of a linear effects model to allow for these repeated observations. Finally, the MUC index has not been validated with the addition of a calprotectin composite score, and future studies may better define and classify IUS activity in UC. The composite noninvasive end point of FC and IUS should be included in future trials to validate and confirm the operating characteristics.

In summary, IUS is a modality that offers immediate, point-of-care assessment in UC that can guide clinical decision-making. Intestinal ultrasound correlates linearly with histological activity, and the sensitivity is enhanced when combined with FC. The role of IUS in a treat-to-target management program will continue to expand as future research better defines activity, response definitions, and treatment targets.

## Supplementary Material

izad043_suppl_Supplementary_Material

## Data Availability

The data that support the findings of this study are available from the corresponding author, (R.V.B.), upon reasonable request.
