# Supplementary material for: Composite Assessment Using Intestinal Ultrasound and Calprotectin Is Accurate in Predicting Histological Activity in Ulcerative Colitis: A Cohort Study
Source: Inflamm Bowel Dis. 2023 Mar 16;30(2):190–5. doi: 10.1093/ibd/izad043 (PMC10834160; doi:10.1093/ibd/izad043)
Supplement: izad043_suppl_Supplementary_Material [file izad043_suppl_supplementary_material.docx]

**Title:** Composite assessment using intestinal ultrasound and calprotectin is accurate in predicting histological activity in ulcerative colitis; a cohort study

**Appendix 1. Milan Ultrasound Criteria**

MUC = 1.4*(colonic wall thickness in mm) + 2*(Colonic wall flow)

Colonic wall flow is graded as 0=absence, 1=presence of blood flow signals at color Doppler.

**Appendix 2. Nancy Index**

Chronic inflammatory infiltrate

0 = no or mild increase

1 = moderate or marked increase that is easily apparent

Acute inflammatory cell infiltrate

2 = mild increase (few or rare neutrophils in lamina propria or in the epithelium that are difficult to see)

3 = moderate or severe increase (presence of multiple clusters of neutrophils in lamina propria and/or in epithelium that are easily apparent)

Ulceration:

4 = severely active disease ((defined by the loss of colonic crypts replaced with ‘immature’ granulation tissue [defined as disorganized blood vessels with extravasated neutrophils] or the presence of fibrinopurulent exudate)

**Appendix 3. Binary comparison tables**

Histological activity (NHI>1) and elevated fecal calprotectin (>50ug/g). Fishers exact test (two sided) p=0.127.

|  | Histologically active | Histologically inactive |
| --- | --- | --- |
| Calprotectin positive | 22 | 3 |
| Calprotectin negative | 2 | 2 |

Histological activity (NHI>1) and elevated fecal calprotectin (>100ug/g). Fishers exact test (two sided) p=0.009.

|  | Histologically active | Histologically inactive |
| --- | --- | --- |
| Calprotectin positive | 19 | 1 |
| Calprotectin negative | 5 | 4 |

Histological activity (NHI>1) and sonographic bowel wall thickness > 3mm. Fishers exact test (two sided) p=0.042.

|  | Histologically active | Histologically inactive |
| --- | --- | --- |
| Bowel wall thickness > 3mm | 14 | 0 |
| Bowel wall thickness ≤ 3mm | 10 | 5 |

Histological activity (NHI>1) and Intestinal Ultrasound activity (Milan Ultrasound Criteria > 6.3). Fishers exact test (two sided) p=0.027.

|  | Histologically active | Histologically inactive |
| --- | --- | --- |
| IUS active | 13 | 0 |
| IUS negative | 11 | 5 |

Histological activity (NHI>1) and composite of Intestinal Ultrasound activity (Milan Ultrasound Criteria > 6.3) and elevated fecal calprotectin (>100ug/g). Fishers exact test (two sided) p=0.001.

|  | Histologically active | Histologically inactive |
| --- | --- | --- |
| Composite positive | 21 | 1 |
| Composite negative | 3 | 4 |
